# Epigenetic inhibition of miR-663b by long non-coding RNA HOTAIR promotes pancreatic cancer cell proliferation via up-regulation of insulin-like growth factor 2

**DOI:** 10.18632/oncotarget.13490

**Published:** 2016-11-22

**Authors:** Huihua Cai, Yong An, Xuemin Chen, Donglin Sun, Tongbing Chen, Yan Peng, Feng Zhu, Yong Jiang, Xiaozhou He

**Affiliations:** ^1^ Department of Hepatobiliary Surgery, The First People's Hospital of Changzhou, The Third Hospital Affiliated to Soochow University, Changzhou, Jiangsu, China; ^2^ Department of Urology, The First People's Hospital of Changzhou, The Third Hospital Affiliated to Soochow University, Changzhou, Jiangsu, China; ^3^ Department of Pathology, The First People's Hospital of Changzhou, The Third Hospital Affiliated to Soochow University, Changzhou, Jiangsu, China

**Keywords:** pancreatic cancer, miR-663b, IGF2, HOTAIR, tumor growth

## Abstract

Pancreatic cancer is one of the most deadly cancers with a poor prognosis. Although microRNAs are involving in the carcinogenesis and development of pancreatic cancer, little information is known regarding the role of miR-663b in pancreatic cancer. In this study, the expression of miR-663b in pancreatic cancer cells was down-regulated by hypermethylation in its putative promoter region, and overexpression of miR-663b repressed cell proliferation, invasion and migration, and induced apoptosis in pancreatic cancer cells. Bioinformatics analysis, luciferase report assay and rescue experiments showed that insulin-like growth factor 2 (IGF2) was a direct target of miR-663b. Results from clinical samples showed that the expression level of miR-663b correlated with the pathological grading, and the expression of miR-663b was down-regulated and was inversely correlated with IGF2 expression level in pancreatic cancer tissues. More importantly, the long non-coding RNA, HOX transcript antisense RNA (HOTAIR), was up-regulated in both pancreatic cancer cells and tissues, and HOTAIR suppressed the expression of miR-663b in pancreatic cancer by histone modification on H3K4me3 and H3K27me3 on miR-663b promoter. Further *in vivo* studies demonstrated that the stable overexpression of miR-663b or knock-down of HOTAIR inhibited tumor growth and was associated with IGF2 expression. In summary, our studies demonstrated that miR-663b is epigenetically repressed by HOTAIR and exerts its tumor-suppressive function via targeting IGF2 in pancreatic cancer.

## INTRODUCTION

Pancreatic cancer is one of the most deadly cancers with the poorest overall 5-year survival rate (<5%) among all human caner types, and the median survival rate of pancreatic cancer is only 6 months [1, 2]. Genetic abnormalities have been regarded as important factors in the development of pancreatic cancer [3]. In addition, current studies demonstrated that epigenetic modifications such as DNA methylation, microRNA (miRNA) deregulation and alteration of long non-coding RNAs (lncRNAs) are considered to have significant impact on the pathogenesis of pancreatic cancer [4–8]. However, their underling molecular mechanisms are largely unknown.

MiRNA is a class of short non-coding RNAs, and it can complement with the 3’ untranslated region (3’UTR) of mRNA, which subsequently represses the expression of targeted genes [9]. The oncogenic or tumor suppressive roles of miRNAs in various types of cancers have been revealed in amounts of studies. For example, miR-93 promotes oncogenesis of cervical cancer by targeting RBA11 family interacting protein1 [10]; miR-143-3p functions as a tumor suppressor in esophageal squamous cell carcinoma via targeting Quaking I-5 [11]; miR-138 suppresses cell proliferation and invasion in hepatocellular carcinoma by inhibiting SOX9 [12]. More importantly, our previous results demonstrated that miR-615-5p was abnormally down-regulated in pancreatic cancer cells due to promoter hypermethylation, which limited its inhibition of insulin-like growth factor 2 (IGF2) and other target genes, thereby contributing to the development of pancreatic cancer [13].

IGF2 is the predominant IGF in adult humans, and IGF2 abnormalities have been associated with a variety of tumors and overexpression of IGF2 is closely related to worse prognosis in cancer. For instance, overexpression of IGF2 promotes cell proliferation and invasion in colorectal cancer [14]; high IGF2 expression is associated with poor clinical outcome in human ovarian cancer [15]. In pancreatic cancer, deregulation of IGF2 has also been shown to be associated with beta-cell tumorigenesis, and pancreatoblastoma is associated with chromosome 11p loss of heterozygosity and IGF2 overexpression [13, 16]. However, the role of IGF2 in pancreatic cancer remains unclear.

LncRNAs are transcripts longer than 200 nucleotides, and they have emerged as important non-coding RNAs in the regulation of gene expression, and deregulation of lncRNAs has been shown to play important roles in various types of cancer including breast cancer, colorectal cancer, non-small cell lung cancer, liver cancer [17–20]. Based on previous studies in pancreatic cancer, several lncRNAs including plasmacytoma variant translocation 1, metastasis-associated lung adenocarcinoma transcript 1, H19, HOTAIR, and HOTTIP exhibit pro-oncogenic activities and correlate with unfavorable outcomes [8]. Gene set enrichment analysis suggests that HOTAIR regulates genes sets mainly associated with cell cycle progression and cell proliferation, and down-regulation of HOTAIR results in a decrease in pancreatic cancer cell proliferation [7]. HOTAIR has been shown to regulated gene expression by recruiting chromatin modifiers. The 5’ end and 3’ end of HOTAIR can bind to polycomb repressive complex 2 (PRC2) and lysine (K)-specific demethylase 1A (LSD1) complex, which allows HOTAIR to bind to a histone methylase and demethylase [21, 22]. Thus, HOTAIR functions as a scaffold for multiple histone modification complexes. Furthermore, studies showed that miRNAs could be regulated by HOTAIR. For example, HOTAIR functions as a competing endogenous RNA to regulate HER2 expression by sponging miR-331-3p in gastric cancer [23]; HOTAIR represses the expression of miR-205 by histone modification in colorectal cancer [24]. Our previous study has showed that several miRNAs were down-regulated due to promoter hypermethylation, and miR-615-5p and miR-663 were well studied in pancreatic cancer [13, 25]. However, the roles of other aberrantly expressed miRNAs such as miR-663b were not fully explored, and whether the interaction exists between HOTAIR and miR-663b is also of great interest to investigate.

In this study, we further explored the role of miR-663b in pancreatic cancer based on our previous study. *In vitro* mechanistic studies revealed the tumor suppressive role of miR-663b via targeting IGF2 in pancreatic cancer, and further study showed that HOTAIR-mediated down-regulation of miR-663b was via regulating histone modification. Further *in vivo* and clinical results confirmed the roles of miR-663b in pancreatic cancer. Therefore, our results may provide some insights into the understanding molecular mechanisms of miR-663b in pancreatic cancer, which could be helpful for the development of new therapeutic target for the treatment of pancreatic cancer.

## RESULTS

### The down-regulation of miR-663b in the pancreatic cancer cells

**Figure 1:**
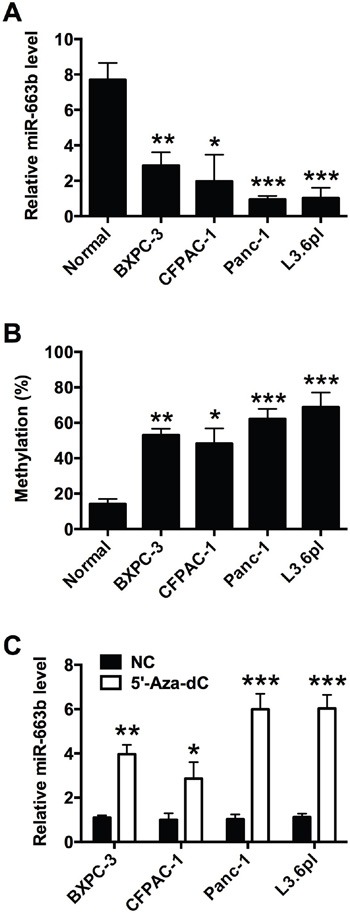
MiR-663b was down-regulated by CpG hypermethylation in pancreatic cancer cells **A**. qRT-PCR analysis of miR-663b expression levels in normal pancreatic tissues from non-cancerous patients and pancreatic cancer cell lines (BXPC-3, CFPAC-1, Panc-1 and L3.6pl). N = 3, significant differences compared to normal pancreatic tissue from non-cancerous patients group were shown as *P<0.05, **P<0.01, ***P<0.001 (One-way ANOVA followed by Dunnett's test). **B**. Methylation status of miR-663b in normal pancreatic tissues from non-cancerous patients and pancreatic cancer cell lines. N = 3, significant differences compared to normal pancreatic tissue group were shown as *P<0.05, **P<0.01, ***P<0.001 (One-way ANOVA followed by Dunnett's test). **C**. qRT-PCR analysis of miR-663b expression levels in pancreatic cell lines treated with 5’-Aza-dc or without 5’-Aza-dc. NC = negative control, n = 3, significant differences compared to NC group were shown as *P<0.05, **P<0.01, ***P<0.001 (Unpaired t test).

To determine the levels of miR-663b in normal pancreatic tissues from non-cancerous patients and pancreatic cancer cell lines, total RNAs were extracted from different types of pancreatic cells, and the miR-663b levels were measured by qRT-PCR. As shown in Figure 1A, the miR-663b levels in pancreatic cancer cell lines (BXPC-3, CFPAC-1, Panc-1 and L3.6pl) were significantly lower than that in normal pancreatic tissues from non-cancerous patients. As our previous study has demonstrated the CpG hypermethylation of miR-663b in pancreatic cancer tissues [13], in the present study, the methylation status of different types of pancreatic cell lines were further determined, and the bisulfite sequencing results showed that the all the pancreatic cancer cell lines were hypermethylated when compared to normal pancreatic tissues (Figure 1B). Treatment with the demethylating agent 5′-Aza-dC significantly increased the expression levels of miR-663b in pancreatic cancer cells when compared to those without 5′-Aza-dC treatment (Figure 1C). Collectively, these results suggest that miR-663b was silenced in pancreatic cancer cell lines by hypermethylation.

### Effect of miR-663b on pancreatic cancer cell proliferation, invasion and migration

**Figure 2:**
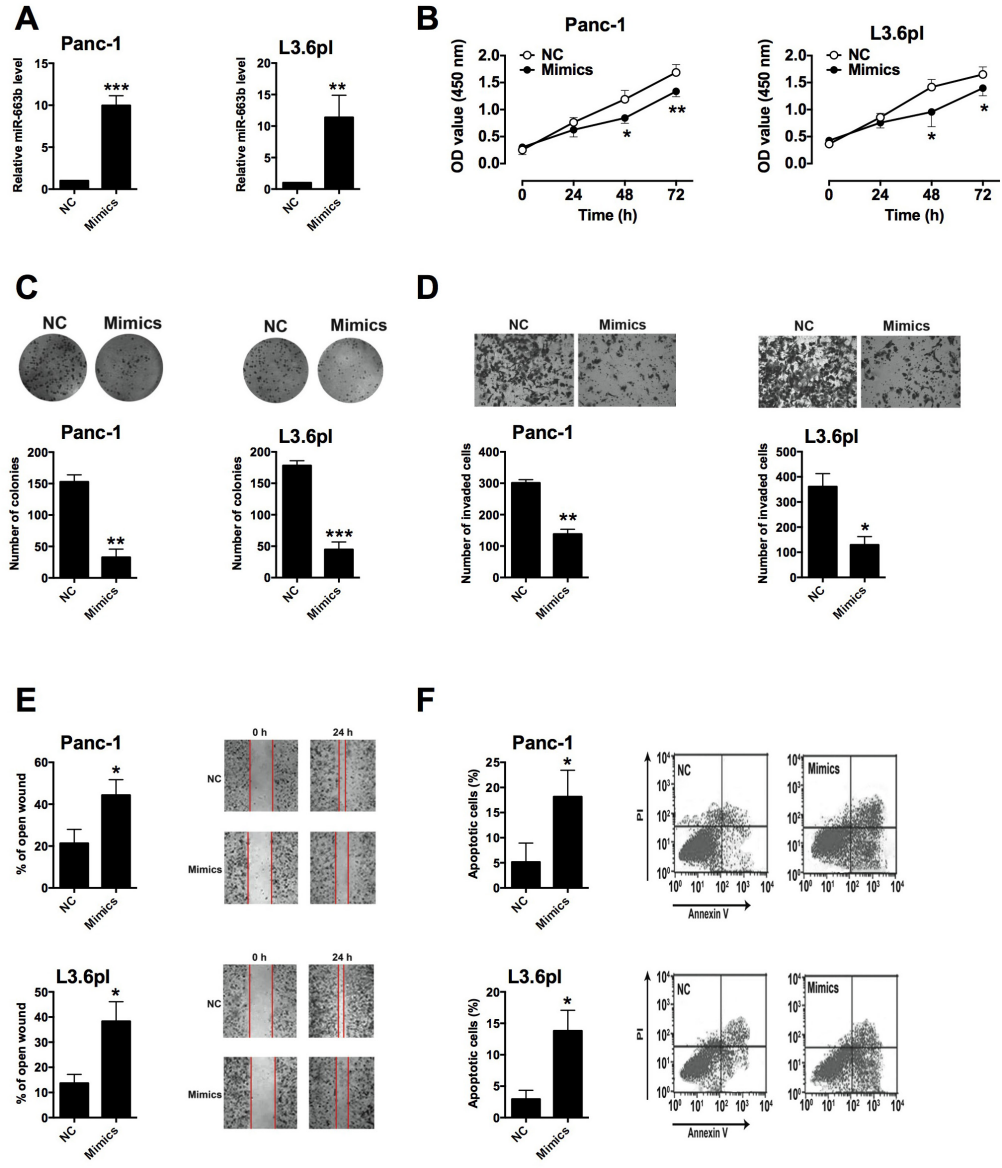
Up-regulation of miR-663b inhibited cell proliferation, invasion and migration in pancreatic cancer cells **A**. qRT-PCR analysis of miR-663b expression levels in pancreatic cancer cells (Panc-1 and L3.6pl) after miR-663b mimics transfection. NC = negative control (scramble miRNAs), mimics = miR-663b mimics, n = 3, significant difference between groups were shown as **P<0.01, ***P<0.001 (Unpaired t-test). **B**. Cell proliferation of pancreatic cells (Panc-1 and L3.6pl) after miR-663b mimics transfection was determined by CCK-8 assay. N = 3, significant differences compared to NC group were shown as *P<0.05, **P<0.01 (Two-way ANOVA followed by Bonferroni's test). **C**. Cell growth, **D**. cell invasion, and **E**. cell migration of pancreatic cells (Panc-1 and L3.6pl) after miR-663b mimics transfection was measured by colony formation assay, Transwell assay, and wound healing assay, respectively. N = 3, significant differences compared to NC group were shown as *P<0.05, **P<0.01, ***P<0.001 (Unpaired t test). **F**. Cell apoptosis of pancreatic cells (Panc-1 and L3.6pl) after miR-663b mimic transfection was analyzed by flow cytometry. N = 3, significant differences compared to NC group were shown as *P<0.05 (Unpaired t test).

To determine the *in vitro* functional role of miR-663b in pancreatic cancer, Panc-1 and L3.6pl cells were transiently transfected with miR-663b mimics or scramble miRNA. QRT-PCR results showed that miR-663b mimics transfection significantly increased miR-663b level in Panc-1 and L3.6pl cells when compared to scramble miRNA transfection (Figure 2A). CCK-8 assay showed that miR-663b significantly reduced cell proliferation in Panc-1 and L3.6pl cells (Figure 2B). The colony formation assay revealed that miR-663b mimics transfection inhibited colony formation in Panc-1 and L3.6pl cells when compared to scramble miRNA transfection (Figure 2C). The invasive ability of Panc-1 and L3.6pl cells as measured by Transwell assay were significantly suppressed by miR-663b mimics transfection (Figure 2D). The wound healing assay demonstrated that overexpression of miR-663b also inhibited the migratory ability of Panc-1 and L3.6pl cells (Figure 2E). Further flow cytometry analysis showed that miR-663b mimics transfection induced apoptosis in Panc-1 and L3.6pl cells (Figure 2F). In summary, these results suggested that overexpression of miR-663b inhibited cell proliferation, invasion and migration, and also induce apoptosis in pancreatic cancer cells.

### MiR-663b repressed IGF2 expression via targeting its 3’UTR

**Figure 3:**
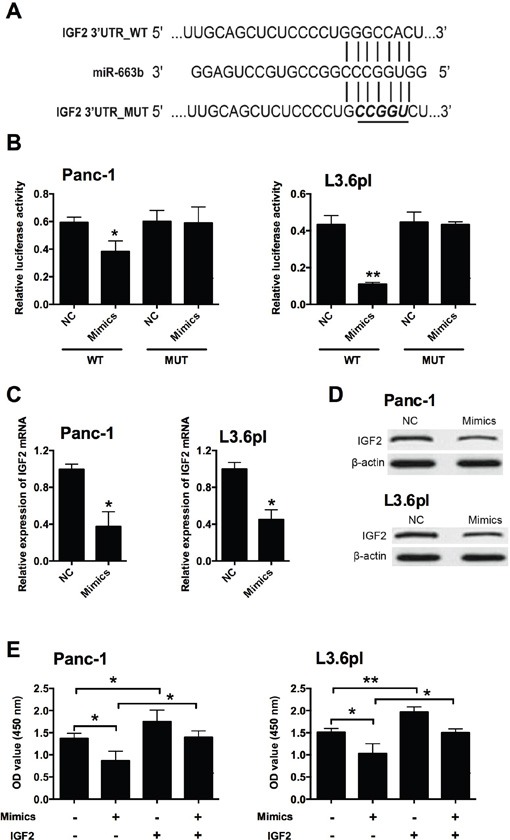
IGF2 was a downstream target of miR-663b in pancreatic cancer cells **A**. Putative miR-663b-binding sequences in the 3’UTR of IGF2 and the reporter constructs showing the wild type (WT) IGF2 3’UTR sequence and the mutated (MUT) IGF2 3’UTR sequence. **B**. miR-663b mimics suppressed the luciferase activity of the WT but not MUT 3’UTR of IGF2 reporter in pancreatic cells (Panc-1 and L3.6pl). NC = negative control (scramble miRNAs), mimics = miR-663b mimics, n = 3, significant differences compared to NC group were shown as *P<0.05, **P<0.01 (Unpaired t test). **C**. qRT-PCR analysis of IGF2 mRNA levels in pancreatic cells (Panc-1 and L3.6pl) after miR-663b mimics transfection. N = 3, significant differences compared to NC group were shown as *P<0.05 (Unpaired t test). **D**. Western blotting analysis of IGF2 protein levels in pancreatic cells (Panc-1 and L3.6pl) after miR-663b mimics transfection. N = 3. **E**. Cell proliferation of pancreatic cells (Panc-1 and L3.6pl) co-transfected with miR-663b mimics (or scrambled miRs) and pcDNA3.1-IGF2 (or pcDNA3.1 vector) was determined by CCK-8 assay. Mimics = miR-663b mimics, IGF2 = pcDNA3.1-IGF2, n = 3, significant differences among groups were shown as *P<0.05, **P<0.01 (One-way ANOVA followed by Dunnett's test).

In order to determine the downstream target of miR-663b, bioinformatics analysis was performed by using Targetscan to predict the potential targets of miR-663b, and IGF2 was predicted as one of the targets of miR-663b (Figure 3A). To confirm whether IGF2 is a target of miR-663b in Panc-1 and L3.6pl cells, luciferase reporter plasmids carrying the wide type (WT) 3’UTR of IGF2 or mutated (MUT) 3’ UTR of IGF2 were constructed. Overexpression of miR-663b markedly inhibited the luciferase activity in the WT 3’UTR of IGF2 in Panc-1 and L3.6pl cells, while luciferase activity was not affected by miR-663b mimics transfection in cells with MUT IGF2 3’UTR reporter (Figure 3B). QRT-PCR analysis showed that miR-663b mimics transfection significantly reduced the mRNA and protein expression levels of IGF2 in Panc-1 and L3.6pl cells when compared to scramble miRNA transfection (Figure 3C and 3D). Further rescue experiments showed that IGF-2 overexpression promoted cell proliferation as measured by CCK-8 assay in Panc-1 and L3.6pl cells when compared to empty vector transfection, and IGF2 overexpression also prevented the reduction in cell proliferative ability caused by miR-663b mimics in Panc-1 and L3.6pl cells (Figure 3E). Therefore, miR-663b down-regulated the transcriptional activity of IGF2, which results in the suppression of cell proliferation.

### MiR-663b level was inversely correlated with IGF2 level in pancreatic cancer tissues

**Figure 4:**
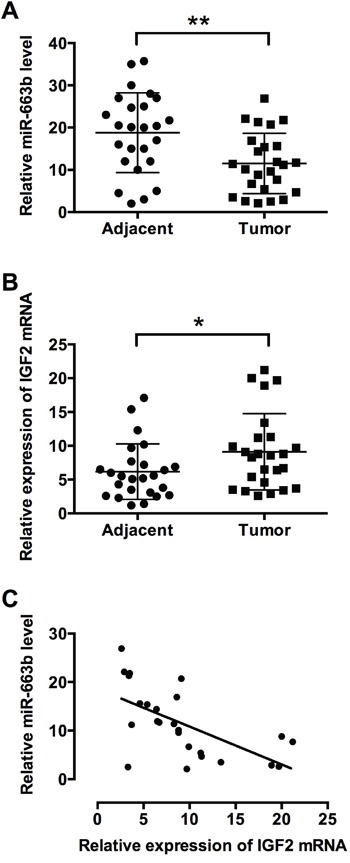
MiR-663b level was inversely correlated with IGF2 level in pancreatic cancer tissues qRT-PCR analysis of **A**. miR-663b levels and **B**. IGF2 mRNA levels in adjacent normal pancreatic tissues and pancreatic cancer tissues from patients with pancreatic cancer. N = 25, significant difference between groups was shown as *P<0.05, **P<0.01 (Paired t test). **C**. The inverse correlation between miR-663b and IGF-2 levels were analyzed by Pearson's correlation test (R = -0.6145, P = 0.0011).

To further confirm the role of miR-663b and IGF2, miR-663b and IGF2 mRNA expression levels were examined in clinical samples of the pancreatic cancer tissues. The clinicopathological analysis of 25 patient tissues showed that there was no significant correlation between miR-663b and age, gender or tumor size, while lower expression level of miR-663b was significantly associated with tumor differentiation, TNM stage and lymph node metastasis (Supplementary Table S1). QRT-PCR results showed that miR-663b expression was significantly lower in pancreatic cancer tissues than that in adjacent normal pancreatic tissues from patients with pancreatic cancer (Figure 4A); while the mRNA expression level of IGF2 was significantly increased in pancreatic cancer tissues when compared to adjacent normal pancreatic tissues from patients with pancreatic cancer (Figure 4B). Pearson's correlation analysis showed that miR-663b levels was inversely correlated with IGF2 level in pancreatic cancer tissues (Figure 4C).

### Effect of HOTAIR on the miR-663b levels in pancreatic cancer cells

**Figure 5:**
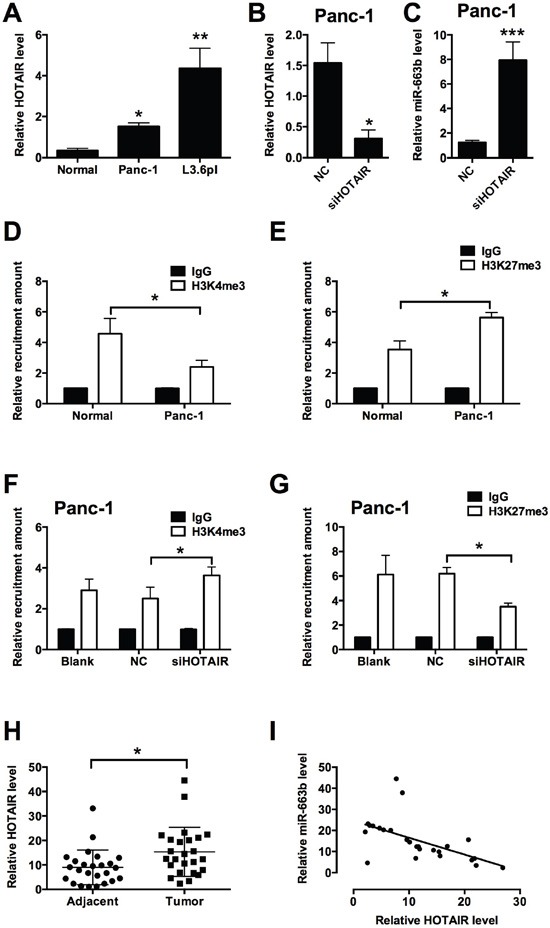
HOTAIR repressed miR-663b level via histone modification in pancreatic cancer **A**. qRT-PCR analysis of HOTAIR level in normal pancreatic tissues and in pancreatic cancer cells (Panc-1 and L3.6pl). N = 3, significant differences compared to normal pancreatic tissues from non-cancerous patients group were shown as *P<0.05, **P<0.01 (One-way ANOVA followed by Dunnett's test). **B**. qRT-PCR analysis of HOTAIR level in Panc-1 transfected with siHOTAIR. NC = negative control, n = 3, significant difference compared to NC group was shown as *P<0.05 (Unpaired t test). **C**. qRT-PCR analysis of miR-663b level in Panc-1 transfected with siHOTAIR. N = 3, significant difference compared to NC group was shown as ***P<0.001 (Unpaired t test). **D** and **E**. ChIP assay analysis showed the recruitment levels of H3K4me3 and H3K27me3 at the promoter region of miR-663b in normal pancreatic tissues from non-cancerous patients and in Panc-1 cells. N = 3, significant differences between groups were shown as *P<0.05 (Unpaired t test). **F** and **G**. ChIP assay analysis showed the recruitment levels of H3K4me3 and H3K27me3 at the promoter region of miR-663b in Panc-1 cells without treatment (Blank) or transfected with scrambled siRNA (NC) or siHOTAIR. N = 3, significant differences between groups were shown as *P<0.05 (Unpaired t test). **H**. qRT-PCR analysis of HOTAIR levels in adjacent normal pancreatic tissues and pancreatic cancer tissues from patients with pancreatic cancer. N = 25, significant difference between groups was shown as *P<0.05 (Paired t test). **I**. The inverse correlation between HOTAIR and miR663b levels were analyzed by Pearson's correlation test (R = -0.5650, P = 0.0037).

HOTAIR has been found to regulate miRNA levels in different types of cancers, and in the present study, the effect of HOTAIR on the miR-663b level was further explored in pancreatic cancer cells. As shown in Figure 5A, the expression level of HOTAIR was up-regulated in Panc-1 and L3.6pl cells when compared to normal pancreatic tissues from non-cancerous patients (Figure 5A). Knock-down of HOTAIR by siHOTAIR transfection significantly reduced the HOTAIR level, while miR-663b level was markedly increased after HOTAIR knock-down (Figure 5B and 5C). Epigenetic modifications, especially methylation at specific histone sites by HOTAIR has an important role in the regulation of miRNA expression levels. The chromatin immunoprecipitation (ChIP) assay results showed that the levels of H3K4me3 were significantly decreased and the levels of H3k27me3 were significantly increased in Panc-1 cells when compared to normal pancreatic tissues from non-cancerous patients (Figure 5D and 5E), while there was no significant difference in the levels of EZH2 and LSD1 between normal pancreatic tissues from non-cancerous patients and Panc-1 cells (data not shown). When the HOTAIR was knocked down in Panc-1 cells, the recruitment of H3K4me3 was increased and the recruitment of H3K27me3 was decreased in Panc-1 transfected with siHOTAIR cells when compared to blank control (Panc-1 cells without treatment) and Panc-1 cells transfected with scramble RNA as negative control (Figure 5F and 5G). In the clinical samples, the levels of HOTAIR were significantly higher in pancreatic cancer tissues than that in adjacent normal pancreatic tissues from patients with pancreatic cancer (Figure 5H); Pearson's correlation analysis showed that HOTAIR level was negative correlated with miR-663b level in pancreatic cancer tissues (Figure 5I). In summary, these results suggest that HOTAIR down-regulated miR-663b via histone modification in pancreatic cancer.

### The effect of miR-663b and HOTAIR on the xenograft tumor growth *in vivo*

**Figure 6:**
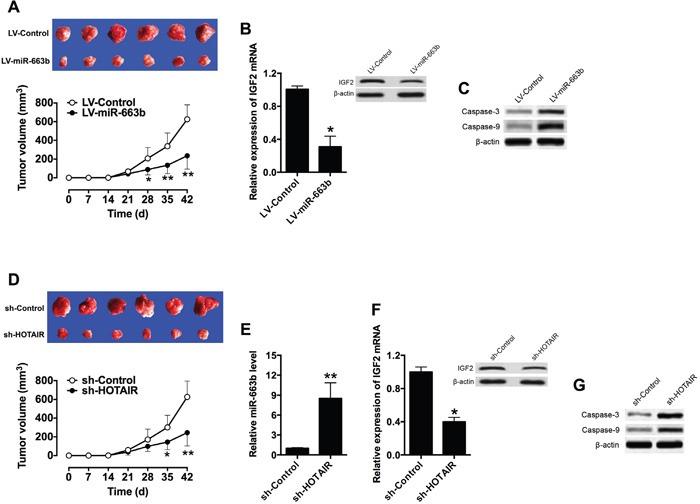
Overexpression of miR-663b or knockdown of HOTAIR suppressed xenograft tumor growth in vivo **A**. Representative images and tumor volume changes in the mice bearing Panc-1 cells with miR-663b or scramble miRNAs. N = 6, significant difference between groups were shown as *P<0.05, **P<0.01 (Two-way ANOVA followed by Bonferroni's test). **B**. qRT-PCR and western blotting analysis of IGF2 mRNA and protein levels in the tumor tissues isolated from the mice bearing Panc-1 cells with miR-663b or scramble miRNAs. N = 6, significant difference between groups was shown as *P<0.05 (Unpaired t test). **C**. Western blotting analysis of caspase-3 and casase-9 protein levels in the tumor tissues isolate from the mice bearing Panc-1 cells with miR-663b or scramble miRNAs. **D**. Representative images and tumor volume changes in the mice bearing Panc-1 cells with sh-HOTAIR or sh-Control. N = 6, significant difference between groups were shown as *P<0.05, **P<0.01 (Two-way ANOVA followed by Bonferroni's test). **E**. qRT-PCR analysis of miR-663b levels in the tumor tissues isolated from the mice bearing Panc-1 cells with sh-HOTAIR or sh-Control. N = 6, significant difference between groups was shown as *P<0.01 (Unpaired t test). **F**. qRT-PCR and western blotting analysis of IGF2 mRNA and protein levels in the tumor tissues isolated from the mice bearing Panc-1 cells with sh-HOTAIR or sh-Control. N = 6, significant difference between groups was shown as *P<0.05 (Unpaired t test). **G**. Western blotting analysis of Caspase-3 and Caspase-9 protein levels in the tumor tissues isolated from the mice bearing Panc-1 cells with sh-HOTAIR or sh-Control.

To examine the functional role of miR-663b and HOTAIR *in vivo*, a xenograft tumor model in nude mice bearing with LV-miR-663b- or sh-HOTAIR-transfected Panc-1 cells was established. The tumor growth was significantly suppressed in the LV-miR-663b group when compared to that in LV-Control group (Figure 6A), and the mRNA and protein levels of IGF2 were also down-regulated in LV-miR-663b group (Figure 6B). The protein levels of factors mediated apoptosis were also examined by western blotting, and the protein levels of caspase-3 and caspase-9 were both up-regulated in LV-miR-633b group (Figure 6C). Moreover, the tumor growth was also suppressed in the sh-HOTAIR group when compared to that in sh-Control group (Figure 6D), and sh-HOTAIR group had higher expression level of miR-663b in the tumor than that in sh-Control group (Figure 6E); also the mRNA and protein levels of IGF2 were down-regulated in sh-HOTAIR group (Figure 6F). The protein levels of caspase-3 and caspase-9 were both up-regulated in sh-HOTAIR group when compared to sh-Control group (Figure 6G). Collectively, these results indicate that miR-663b inhibited the proliferative ability of Panc-1 *in vivo*, and this inhibitor effect may be associated with HOTAIR.

## DISCUSSION

In this study, we demonstrated that miR-663b inhibited cell proliferation, invasion and migration in pancreatic cancer cells. Bioinformatics analysis, luciferase report assay, and rescue experiment revealed that IGF2 was a novel target of miR-663b. Furthermore, HOTAIR was found to suppress the expression level of miR-663b in pancreatic cancer cells by modifying histone methylation on miR-663b promoter. The effects of miR-663b on pancreatic cancer development were further confirmed in the *in vivo* studies and in clinical samples.

Aberrant expression of miRNAs has been closely correlated with tumorigenesis. In pancreatic cancer, abundant miRNAs have been identified either as oncogenic miRNAs or tumor-suppressive miRNAs. The role of miR-663b was largely unknown in cancer progression. In the colorectal cancer, miR-663b was one of the important miRNAs to differentiate between colorectal carcinoma and normal colonic mucosa [26]; miR-663b was down-regulated in bladder cancer plasma and has been suggested as a potential biomarker for bladder cancer [27]; miR-663b was also found to be dysregulated in human gastric cancer [28]. In our previous study, we identified several aberrantly expressed miRNAs in pancreatic cancer tissues by using methylated DNA immunoprecipitation-chip analysis, and miR-615-5p was found to be epigenetically inactivated and functions as a tumor suppressor in pancreatic cancer. MiR-663b was also found to be hypermethylated in pancreatic cancer cells [13]. In this regard, we further explored the role of miR-663b in pancreatic cancer. Our results demonstrated miR-663b was inactivated in pancreatic cancer cell lines via CpG island hypermethylation. Further *in vitro* functional study demonstrated that overexpression of miR-663b suppressed cell proliferation, invasion and migration and also induced cell apoptosis in pancreatic cancer cells; *in vivo* study revealed that, tumor growth was suppressed in the mice bearing Panc-1 cells with miR-663b. More importantly, the expression level of miR-663b correlated with the pathological grading and was down-regulated in pancreatic cancer. Therefore, these results suggest that miR-663b functions as a tumor suppressor in pancreatic cancer.

By using informatics analysis, IGF2 was found to be a downstream target of miR-663b. IGF2 is the predominant IGF in adult humans, and IGF2 abnormalities have been associated with a variety of tumors and overexpression of IGF2 is closely related to worse prognosis in cancer. Deregulation of IGF2 has shown to be associated with beta-cell tumorigenesis, and pancreatoblastoma is associated with chromosome 11p loss of heterozygosity and IGF2 overexpression [14, 15]. Further, our previous study showed that IGF2 was down-regulated in pancreatic cancer tissues and was a direct target of miR-615-5p. Luciferase reporter assay and rescue experiment confirmed that miR-663b targets 3’UTR of IGF2 to suppress the expression of IGF2 and inhibits pancreatic cancer cell proliferation. In the clinical samples, IGF2 was up-regulated in pancreatic cancer tissues, and miR-663b level was inversely correlated with IGF2 level in the pancreatic cancer. More importantly, the *in vivo* functional study showed that miR-663b inhibited tumor growth and repressed IGF2 expression. Therefore, these results may suggest that miR-663b targets IGF2 to regulate the tumor progression in pancreatic cancer. However, we should be cautious that miR-663b may targets more than just one downstream targets to exert its function, which may require further investigation.

Human HOTAIR, is a 2.2 kb lncRNA transcribed from the HOXC locus and has been shown to bind both to the PRC2 and the LSD1 complexes, which allows HOTAIR to function as a scaffold for multiple histone modification complexes [22]. Aberrant expression of HOTAIR has been associated with tumorigenesis in various types of cancer including breast cancer, colorectal cancer, non-small cell lung cancer, liver cancer [13, 18–20]. However, the functional role of HOTAIR is largely still unclear in pancreatic cancer. In this study, HOTAIR was found to be up-regulated in both pancreatic cancer cell lines and pancreatic cancer tissues, and HOTAIR level was inversely correlated with miR-663b level in pancreatic cancer tissues. Moreover, HOTAIR disrupted the balance between H3K4me3 and H3K27me3 on the miR-663b promoter region, which may be associated with the hypermethylation status of miR-663b in the pancreatic cancer cells [29, 30]; thus HOTAIR suppressed the expression of miR-663b in pancreatic cancer cells. Knock-down of HOTAIR in Panc-1cells suppressed the *in vivo* tumor growth, suggesting that the *in vivo* tumor suppressive effect of miR-663b may be associated with HOTAIR. Collectively, these results suggest that the interaction between miR-663b and HOTAIR is key for the pancreatic cancer development.

In conclusion, our studies demonstrated that miR-663b is epigenetically regulated by HOTAIR and exerts its tumor-suppressive function via targeting IGF2. Our findings suggest that miR-663b may be an important diagnostic marker or potential therapeutic target for the treatment of pancreatic cancer.

## MATERIALS AND METHODS

### Cell culture

The human pancreatic cancer cell lines (BXPC3, CFPAC-1, Panc-1 and L3.6pl) and HEK-293T cells were purchased from the Cell Bank of the Chinese Academy of Sciences (Shanghai, China). Cells were cultured in Dulbecco's modified Eagle's medium (DMEM, Sigma, St Louis, USA) supplemented with 10% fetal bovine serum (HyClone, GE Healthcare Life Science, Logan, USA) and incubated in a humidified chamber supplemented with 5% CO_2_ at 37°C.

### Tissue samples

Pancreatic cancer tissues and their matched adjacent normal pancreatic cancer tissues (approximately 5 cm from cancerous tissues) were taken from 25 patients undergoing surgery for pancreatic cancer at the First People's Hospital of Changzhou, and normal pancreatic control tissues were taken from non-cancerous patients undergoing pancreaticoduodenectomy. All cases were reviewed by pathologist and histologically confirmed as pancreatic cancer based on histopathological evaluation. The characteristics of the patients were shown in Supplementary Table S1. All tissues were immediately snap-frozen in liquid nitrogen and stored at -80°C until further experimentation. No local or systemic treatment was conducted in these patients before surgical operation. Informed consents were obtained from all patients, and the study was approved by the Research Ethics Committee of the First People's Hospital of Changzhou.

### Oligonucleotide transfection, plasmid construction and lentiviral infection

MiR-663b mimics and its negative control, scramble miRNAs were purchased from Ribobio (Guangzhou, China). The IGF2 mRNA sequences were synthesized and subcloned into the pCDNA3.1 vector, and the empty pcDNA3.1 vector served as negative control (Genepharma, Shanghai, China). For HOTAIR, the siRNA specially targeting HOTAIR or its non-target negative control siRNA was synthesized by Genepharma. For transfection, Panc-1 cells or L3.6pl cells were grown on six-well plates to 60% confluence, and miRNA, siRNA or plasmid transfection was performed by using Lipofectamine 2000 (Invitrogen) according to the manufacturer's protocol. For the rescue experiment, cells were co-transfected with miRNAs (miR-663b mimics or scramble miRNA) and plasmids (pcDNA3.1-IGF2 or pcNDA3.1). Total RNA and protein were extracted at 24 h post transfection and used for qRT-PCR and western blot analysis.

MiR-663b-overexpressing lentiviral constructs were generated using synthetic oligonucleotides and the Lv-CMV-GPF vector (Genepharma), and shHOTAIR-overexpressing lentiviral constructs were generated by subcloning shHOTAIR into pGLV3/H1/GFP lentiviral frame plasmids (Genepharma); empty vectors were used as negative controls, respectively. All the constructed plasmids were confirmed by sequencing (Invitrogen, Carlsbad, USA). Lentivirus carrying miR-663b or HOTAIR was packaged in the HEK293T cells and collected from the supernatants following the manufacturer's protocol. Stable cell lines for xenograft study were established by infecting the lentivirus into Panc-1 cells.

### RNA extraction and qRT-PCR analysis

Total RNA was extracted from cells or tissues using TRIZOL reagent (Invitrogen) according to the manufacturer's instruction. IGF2 mRNA level and HOTAIR was quantified by qRT-PCR using a SYBR Premix ExTaq Reverse Transcription PCR kit (Takaka, Dalian, China) and GAPDH was used as an internal control for normalization (see Supplementary Table S2 for primers sequence). For miR-663b detection, the miR-663b level was quantified by qRT-PCR using TaqMan assay kits (Applied Biosystems, Forster City, USA) and U6 was used an internal control for normalization. The primers for qRT-PCR were shown in Supplementary Table S2. The reaction was performed using an ABI PRISM 7500 Sequence Detection System (Applied Biosystems). The relative expression levels of IGF2 mRNA, HOTAIR and miR-663b were calculated using 2^ΔΔCt^ method.

### 5-aza-2’deoxycytidine treatment

Pancreatic cancer cells were cultured overnight, and 5-aza-2’-deoxycytidine (5’-Aza-dC) was added and refreshed every 24 h for 48 h. The medium containing PBS was used as a negative control.

### DNA isolation and methylation analysis

Methylation analysis of miR-663b was performed according to our previous study. Briefly, genomic DNA was extracted from cells or tissues using DNeasy kit (Qiagen, Hilden, Germany). Bisulfite modification of the genomic DNA was performed by sing an Eptitech Bisulfite kit (Qiagen). For bisulfite sequencing, an aliquot of 1 μl of the sodium bisulfite-treated DNA was amplified by PCR. Using the TA cloning kit (Toyobo, Osaka, Japan), 2 μl of the PCR product was cloned into the pTA2 vector. Sequencing analysis was performed on six clones or more for each sample.

### Western blotting

Protein were extracted from cells or tissues using RIPA lysis buffer. Proteins were then separated by 12% SDS-PAGE. After electrophoresis, proteins were transferred onto polyvinylidene difluoride membrane. After blocking with 5% non-fat milk for 2 h at room temperature, the membranes were then incubated with rabbit polyclonal IGF2 antibody (sc-5622; Santa Cruz, Dallas, USA), rabbit polyclonal antibody (#9662; Cell Signaling Technology, Beverly, USA), rabbit polyclonal antibody (#9504, Cell Signaling Technology), or mouse monoclonal β-actin (sc-47778; Santa Cruz) overnight at 4°C. The membranes were then incubated 2 h at room temperature with horseradish peroxidase-conjugated goat anti-rabbit or goat anti-mouse (Santa Cruz) secondary antibody and visualized with a chemiluminescence kit (Pierce, Rockford, USA).

### CCK-8 assay

Cell proliferation assay was performed with Cell Counting Kit-8 (Dojindo, Kumamoto, Japan) according to the manufacturer's instruction. Twenty-four hour after transfection, cells were seed in 96-well plates at 1x10^4^ cells per well. The proliferative ability of cells was determined at 0, 24, 48, and 72 h. For the rescue experiment, cells were co-transfected with miRNAs (miR-663b mimics or scramble miRNA) and plasmids (pcDNA3.1-IGF2 or pcNDA3.1), and the proliferative ability of cells was determined at 48 h. CCK-8 (10 μl) was added to each well at different time points, and the plate was incubated for 2 h at room temperature. The absorbance was measured at 450 nm using a microplate spectrophotometer (Molecular Devices, Sunnyvale, USA).

### Colony formation assay

Twenty-four hour after transfection, cells were seeded for colony formation in 6-cm culture dishes at a density of 1000 cells per dish. After continuous culture for 14 days, cells were fixed in methanol for 10 min and stained with 0.1% crystal violet for 30 min. Visible colonies were manually counted.

### Transwell assay

Transwell assay was performed using a chamber of 6.5 mm in diameter and with an 8-mm pore size (Corning, Corning, USA). Twenty-four hour after transfection, 5 x 10^4^ cells in 0.1% FBS were seeded onto the Matrigel-coated membrane matrix of the upper chamber, and the lower chamber was filled with 10% FBS. After 24 h incubation, cells invading the bottom of the membrane were stained with 0.1% crystal violet in 20% ethanol, and the number of invaded cells was counted by using a DM2500 bright field microscope (LEICA, Wetzlar, Germany).

### Wound healing assay

Twenty-four hour after transfection, a wound was created in adherent cells using a 20 μl pipette tip. The cells then cultured in DMEM without FBS for 24 h, and the images of migrated cells and wound healing were taken by using a DM2500 bright field microscope (LEICA).

### Cell apoptosis analysis

Twenty-four hour after transfection, cells were trypsinized and fixed with 70% ethanol for 30 min on ice. RNA was degraded by incubation with 20 mg/ml RNase (Sigma) for 1 h at 37°C. Cells were stained with FITC-Annexin V and propidum iodide (Beyotime, Beijing, China) and then were analyzed by Calibur flow cytometry (BD Biosciences, Franklin Lakes, USA) equipped with CellQuest software (BD Biosciences).

### Luciferase reporter assay

Cells were seeded in 96-well plates at 1x10^4^ cells per well. When the cells reached 60% confluence, they were co-transfected with wide type (WT) pGL3-IGF2 3’UTR or mutant (MUT) pGL3-IGF2 3’UTR plasmids and either scramble or miR-663b mimics using Lipofectamine 2000 (Invitrogen). Forty-eight hours after transfection, luciferase activity was measured with the Dual-Luciferase Reporter Assay System (Promega, Madison, USA) and expressed as the ratio between firefly and Renilla luciferase activities.

### Chromatin immunoprecipitation (ChIP) assay

Cells were fixed with 4% paraformaldehyde and sonicated to prepare the chromatin fragments. Chromatin samples were immunoprecipitated with following antibodies at 4°C for 3 h: Rabbit anti-EZH2 (Cell Signaling Technology, Danvers, USA), Rabbit anti-LSD1 (Cell Signaling Technology), Rabbit anti-H3K4eme3 (Abcam, Cambridge, USA), H3K27me3 (Abcam), or normal rabbit IgG (Santa Cruz) antibodies. After crossing reversal, precipitated DNA was analyzed by PCR to detect a 167 bp fragment of miR-663b promoter region (see Supplementary Table S2 for primers sequence). The data were calculated by normalizing against that of corresponding DNA precipitated by rabbit IgG.

### *In vivo* animal study

Tumor formation was studied by establishing a xenograft model. Four-week-old BALB/c nude mice were purchased from the Shanghai Experimental Animal Center (Chinese Academy of Sciences). The animal experiments in this study were approved by the Animal Research Committee of the First People's Hospital of Changzhou. Care and handling of the animals were in accordance with the guidelines for Institutional and Animal Care and Use Committees. A total of 24 animals were randomly divided into 4 groups with 6 animals in each group. Infected Panc-1 cells (1×10^6^/mice) were subcutaneously injected into the neck area of the nude mice. Tumor volumes (mm^3^) were measured every 7 days and calculated using the following formula: volume = width × length × height/2. The animals were killed 42 days after cell inoculation, and tumor tissues were harvested for further analysis.

### Statistical analysis

All the data were expressed as mean ± SD. Statistical analysis were performed by using Student's t-test or ANOVA followed by multiple comparison tests. The relationship between the expression of miR-663b, IGF2 and HOTAIR was examined by Pearson's correlation analysis. The correlation between miR-663b and pathological parameters was determined by Chi-square test. Differences were considered to be statistically significant when P<0.05. All the results were performed in at least three independent experiments.

## SUPPLEMENTARY TABLES


