# Supplementary material for: Epigenetic inhibition of miR-663b by long non-coding RNA HOTAIR promotes pancreatic cancer cell proliferation via up-regulation of insulin-like growth factor 2
Source: Oncotarget. 2016 Nov 22;7(52):86857–70. doi: 10.18632/oncotarget.13490 (PMC5349959; doi:10.18632/oncotarget.13490)
Supplement: Supplementary file 1 [file oncotarget-07-86857-s001.pdf]

Epigenetic inhibition of miR-663b by long non-coding RNA HOTAIR promotes pancreatic cancer cell proliferation via up-regulation of insulin-like growth factor 2

SUPPLEMENTARY TABLES

Supplementary Table S1: The association between miR-663b levels and clinicopathological characteristics of pancreatic cancer patients

	miR-663b expression		*P*-value
	Low expression	High expression	
	N=14	N=11	
Age			
<54 years	9	4	0.1654
≥ 54 years	5	7	
Gender			
Male	8	5	0.5615
Female	6	6	
Tumor differentiation			
1-2	3	9	0.0027
3	11	2	
TNM stage			
I-II	2	8	0.0031
III/IV	12	3	
Nodal metastasis			
0	5	9	0.0212
1	9	2	
Tumor size			
<2 cm	3	5	0.2011
>2 cm	11	6	

Low expression and high expression of miR-663b was determined by the cut-off values for miR-663b, which were defined as the cohort median, and median of age was used as the cut-off values to define the subgroup (<54 years old and ≥ 54 years old group). Statistical significance between groups was analyzed by Chi-square tests.

Supplementary Table S2: Primers for PCR experiment

	Forward primers	Reverse primers
miR-663b	5'-TATTTTATTAAGGGGGAAGTGT-3’	5’-CCTCRATAAAAAAACCTTCTCT-3’
IGF2	5’-CTTGGACTTTGAGTCAAATTGG-3’	5’-GGTCGTGCCAATTACATTTCA-3’
GAPDH	5’-AGAAAATCTGGCACCACACC-3’	5’-TAGCACAGCCTGGATAGCAA-3’
U6	5'-CTCGCTTCGGCAGCACA-3'	5'-AACGCTTCACGAATTTGCGT-3'
miR-663b promoter region	5'-TTCGAGGTTAAGAATGGCAG-3'	5'-CACCGCCTTGTAGTCCACTTT-3'
HOTAIR	5'-GGTAGAAAAAGCAACCACGAAGC-3'	5'-ACATAAACCTCTGTCTGTGAGTGCC-3'
